# Micronucleus quantification from whole-slide haematology images using AI serves as a translatable pharmacodynamic biomarker for DNA damage response inhibitors

**DOI:** 10.1038/s41598-026-41458-7

**Published:** 2026-02-28

**Authors:** Killian H. R. Yong, Weronika S. Robak, Lee Mulderrig, Adina Hughes, Richard Bystry, Tanya Wantenaar, Gemma N. Jones, Maria Udriste, Jack Robertson, Josep V. Forment, Lenka Oplustil O’Connor, Ross J. Hill

**Affiliations:** 1 Translational Pathology, Cancer Biomarker Development, Oncology R&D, AstraZeneca, Cambridge, UK; 2 Bioscience, Oncology Targeted Discovery, Oncology R&D, AstraZeneca, Cambridge, UK; 3 Translational Medicine, Oncology R&D, AstraZeneca, Cambridge, UK; 4 Oncology Global Diagnostics, Oncology Business Unit, AstraZeneca, Cambridge, UK

**Keywords:** Biological techniques, Biomarkers, Cancer, Computational biology and bioinformatics

## Abstract

Micronuclei are widely recognised biomarkers of genomic instability and DNA damage, making their accurate quantification essential for understanding the pharmacodynamic properties of chemotherapeutic agents and inhibitors of the DNA damage response (DDR). Here, we report the development and validation of a novel assay for the automated detection and quantification of micronuclei within circulating red blood cells (RBC) from peripheral blood smears. We integrate recent advances in whole-slide imaging (WSI) technologies and supervised deep-learning algorithms to quantify micronuclei in over 100,000 RBCs from a single image. We demonstrate that this approach achieves strong analytical concordance with flow cytometry (Pearson’s r = 0.926, *P* < 0.0001) while offering distinct advantages. Additionally, using May-Grünwald Giemsa dyes we show that deep-learning algorithms can stratify red blood cells into both mature erythrocytes and immature reticulocytes from WSIs. Critically, we establish that micronuclei-positive red blood cell (MN^+^-RBC) frequency correlates with anti-tumor efficacy in *BRCA1*-deficient xenograft models following exposure to PARP inhibitors and demonstrates dose-dependent pharmacodynamic (PD) responses. Furthermore, we show that whole-slide imaging offers several advantages over widely used flow cytometry approaches, including the identification of cells with multiple micronuclei and the ability to quantify morphological features associated with detrimental pre-analytical conditions. These findings position automated WSI-based micronucleus quantification as a scalable, minimally invasive PD biomarker requiring only 5 μl of blood that enables longitudinal monitoring of DDR inhibitor therapies.

**Supplementary Information:**

The online version contains supplementary material available at 10.1038/s41598-026-41458-7.

## Introduction

Micronuclei are small, extranuclear structures that contain either entire chromosomes or chromosomal fragments encapsulated by an atypical nuclear membrane^1^. Micronuclei primarily arise following mitotic errors and the subsequent mis-segregation of lagging chromosomes, or the isolation of acentric chromosomal fragments following unresolved/mis-repaired DNA damage^1^. The genetic material partitioned into micronuclear structures is associated with a defective DNA damage response (DDR), the accumulation of unresolved DNA damage and aberrant DNA replication kinetics^2^. Critically, the atypical nuclear envelope surrounding a micronucleus is prone to rupture, exposing the genetic material sequestered within to the cytosol^3,4^. This rapid and irreversible loss of compartmentalisation is a major event during the aberrant lifecycle of micronuclei and is associated with the accumulation of DNA damage^3^, chromosome shattering^2,5^ and a spectrum of structural DNA rearrangements, including chromothripsis^6,7^. Furthermore, rupture of the micronucleus envelope is linked to epigenetic alterations and changes to chromatin accessibility^8^. Ultimately, while micronuclei may persist for several cellular generations, they can eventually become reincorporated into daughter nuclei along with any genetic and epigenetic alterations^7,8^. Therefore, micronuclei are not merely passive indicators of genomic instability, instead, they actively facilitate complex mutational processes and drive cancer genome evolution through the sequestration of chromosomes in unstable membranous compartments.

Consequently, the quantification of micronuclei has emerged as a powerful biomarker of chromosome instability (CIN), genotoxicity^9^, inherited genomic instability syndromes^10^, ageing^11^ and as a predictive biomarker for both cancer risk and treatment response^12,13^. Indeed, an increased frequency of micronuclei in urothelial or buccal exfoliate cells is associated with cancer risk or exposure to environmental genotoxins in humans^12,14,15^. In the context of cancer therapy, micronucleus quantification provides a direct functional readout of pharmacodynamic (PD) activity for therapies that exploit DNA repair vulnerabilities. Inhibitors of the DNA damage response, such as PARP inhibitors that induce synthetic lethality in homologous-recombination-deficient cancers, lead to increased micronucleus formation^16,17^. Furthermore, micronuclei have recently emerged as a prognostic biomarker for cancer treatment^12,18,19^. However, difficulties in the faithful identification of micronuclei in routine histopathology sections of formalin-fixed paraffin-embedded (FFPE) tumor specimens remains a major barrier to the broad adoption of micronucleus quantification in clinical settings. This is in part due to the challenge of distinguishing *bona fide* micronuclei from partial sections of primary nuclei in adjacent z-plane sections^20^.

Crucially, erythropoiesis, the process by which mature red blood cells (RBCs) are produced, represents a unique cellular differentiation program in which the primary nucleus is extruded during red cell maturation. This process results in immature red blood cells, known as reticulocytes, entering peripheral circulation without a nucleus. Importantly, micronuclei that arise during erythropoiesis are frequently retained following nuclear extrusion, resulting in easily identifiable nuclear remnants in peripheral red blood cells. Indeed, these nuclear remnants were first described in the late 19^th^ century, referred to as Howell-Jolly bodies^21,22^, and were later shown to increase in frequency following exposure to ionising radiation or following splenectomy in humans^9^. Consequently, Howell-Jolly bodies represent easily identifiable *bona fide* micronuclei in peripheral red blood cells that can serve as a readily accessible surrogate system to assess micronucleus formation, circumventing the difficulties of micronucleus identification in complex histological specimens.

Traditionally, micronucleated RBCs are quantified either by flow cytometry using methanol-fixed whole-blood specimens or the manual assessment of peripheral blood films using a traditional brightfield microscope. Both methods are labour-intensive and not readily adaptable to routine clinical workflows. To address these limitations, we developed and validated a novel whole-slide imaging (WSI) approach for automated micronucleus quantification in peripheral red blood cells. Our focus was two-fold. Firstly, we sought to establish this method as a robust PD biomarker through validation against flow cytometry, and the demonstration of dose-dependent responses and kinetics following exposure to PARP inhibitors. Secondly, we aimed to enhance the translatability of micronucleus quantification from WSIs by enabling reticulocyte-specific analysis using standard May-Grünwald Giemsa dyes. This approach provides enhanced temporal resolution for PD monitoring while requiring standard histological stains, rather than specialized fluorescent markers.

By leveraging whole-slide imaging and the development of supervised deep-learning algorithms, we achieve high-throughput analysis of up to 100,000 RBCs per sample from only 5 μl of blood. Using this approach, we demonstrate a PD-efficacy relationship and reveal the kinetics of micronucleus-positive (MN^+^)-RBC induction in the context of olaparib, a clinically approved PARP inhibitor, and saruparib, a new generation PARP1-selective PARP inhibitor^23^. These data position automated WSI-based micronucleus quantification from peripheral blood smears as a scalable, translatable tool for clinical biomarker development and drug response monitoring.

## Results

**Fig. 1:**
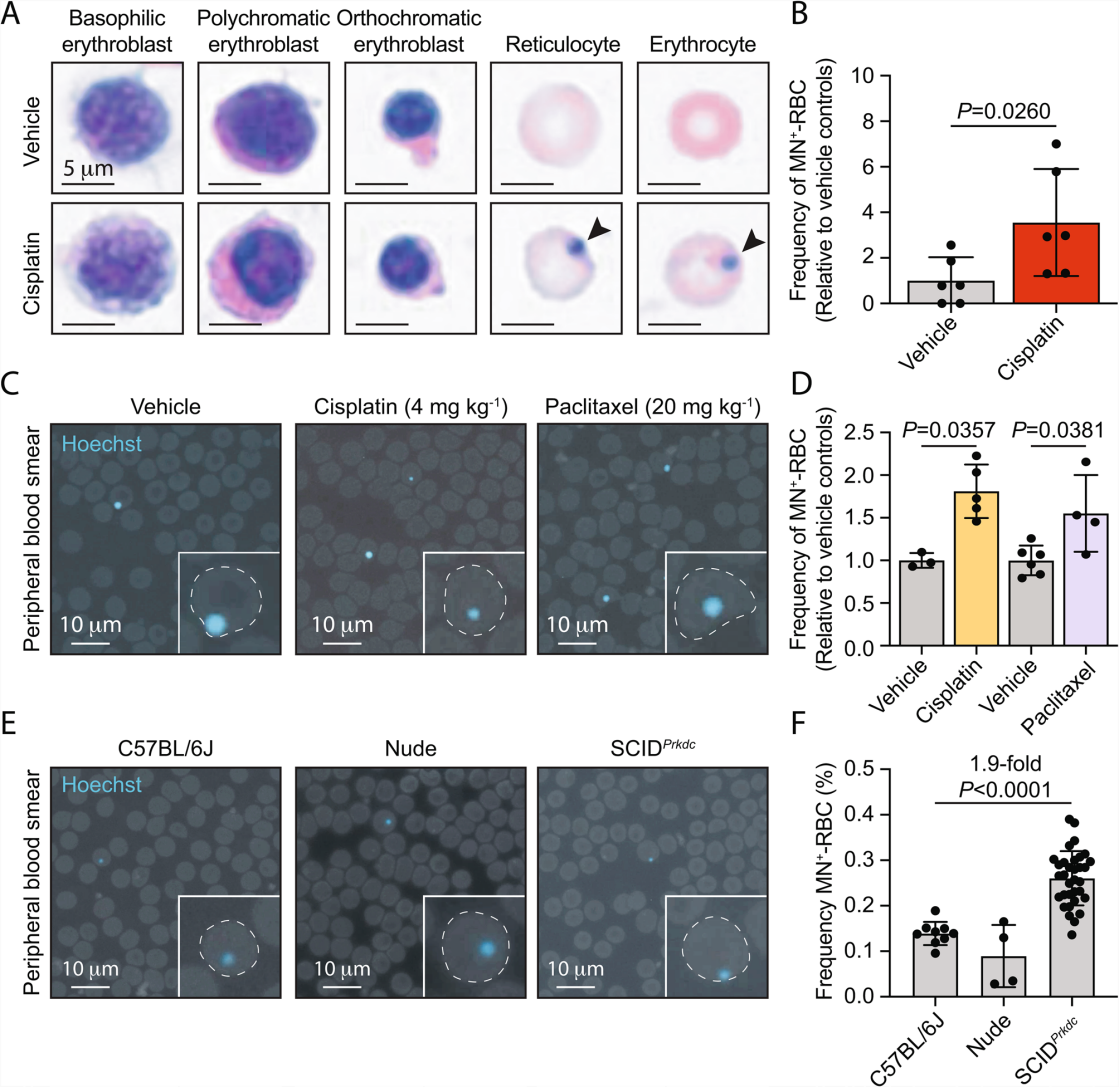
Quantification of micronucleus-positive red blood cells from whole-slide haematology images using supervised deep-learning. **A**, **B**, Representative images of H&E-stained bone marrow aspirate smears from SCID^*Prkdc*^ mice exposed to 4 mg kg^-1^ cisplatin or vehicle-only controls (arrowheads denote micronucleus-positive cells) and the quantification of micronucleus-positive red blood cells (MN^+^-RBCs) (*P*-values calculated using a two-tailed Mann–Whitney *U*-test, data are mean ± s.d.; *n* = 2 aspirates per mouse, 3 mice per treatment group). **C**,**D**, Representative images of Hoechst-stained peripheral blood films collected from mice 48-hours after exposure to either 4 mg kg^-1^ cisplatin, 20 mg kg^-1^ paclitaxel or vehicle-only controls and quantification of the frequency of micronucleated red blood cells (MN^+^-RBCs) (*P*-values calculated using a two-tailed Mann–Whitney *U*-test, data are mean ± s.d.; each point represents data from one mouse, *n* = 3, 5, 6 and 4, left to right)*.* E, F, Representative images of Hoechst-stained peripheral blood films from treatment naive 6-to-8-week-old female C57BL/6, NMR1-*Foxn1*^*nu/nu*^ Nude and SCID^*Prkdc*^ mice and quantification of the frequency of micronucleus-positive red blood cells (MN^+^-RBCs) (*P*-values calculated using a two-tailed Mann–Whitney *U*-test, data are mean ± s.d.; each point represents data from one mouse, *n* = 9, 4 and 37, left to right).

To study the utility of WSIs for the quantification of micronucleated RBCs, we first assessed bone marrow aspirate smears as micronuclei arise within haematopoietic progenitors prior to the entry of RBCs into peripheral circulation. To do this, we acquired WSIs at 40x magnification of bone marrow smears derived from mice exposed to the chemotherapeutic, DNA-damaging agent, cisplatin, or vehicle-only controls and quantified the frequency of MN^+^-RBCs (Fig. 1A). We observed a significant 3.6-fold increase in the frequency of MN^+^-RBCs following exposure to 4 mg kg^-1^ cisplatin compared to vehicle-only controls (Fig. 1B). However, the quantification of MN^+^-RBCs from bone marrow aspirates is made difficult due to the abundance of nucleated erythroid precursors and the presence of orthochromatic erythroblasts undergoing the process of nuclear compaction and expulsion (Fig. 1A). This led us to question if similar WSI techniques could be used to quantify MN^+^-RBCs in peripheral circulation, which offers several translational advantages. Firstly, peripheral blood can be collected more frequently to allow longitudinal analysis. Secondly, peripheral blood collection is significantly less invasive than acquiring bone marrow aspirates. Finally, peripheral RBCs are enucleated, resulting in easily identifiable micronuclei.

To this end, we acquired peripheral blood smears from mice exposed to either cisplatin, or the aneugen, paclitaxel, and stained them with Hoechst 33342, a fluorescent DNA dye, and acquired WSIs at 40x magnification (Fig. 1C). Using commercially available deep-learning tools, we developed supervised U-Net convolutional neural networks (CNNs) to detect and classify RBCs as either micronucleus positive (MN^+^-RBC; containing a Hoechst-positive micronucleus structure) or micronucleus negative (MN^-^-RBC; absent Hoechst-positive micronucleus structure) (Supplementary Figure 1). First, we set out to establish the reproducibility of MN^+^-RBC quantification from WSIs by analysing technical replicates obtained through serial sampling of treatment-naive mice. Specifically, we collected multiple independent samples from individual mice and prepared separate blood smears from each sample to assess intra-animal reproducibility. We observed coefficients of variance of 4.8-10.2% and 19.0-19.2% for samples obtained from C57BL/6J and SCID^*Prkdc*^ mice, respectively (Supplementary Figure 2). Having established technical reproducibility, we next quantified the frequency of MN^+^-RBCs following exposure of mice to either cisplatin or paclitaxel. We observed a significant 1.8 and 1.6-fold induction in the frequency of MN^+^-RBC after exposure to cisplatin (*P* = 0.0357) or paclitaxel (*P* = 0.0381) as compared to vehicle-only controls, respectively (Fig. 1C,D). These data are consistent with the respective clastogenic and aneugenic properties of cisplatin and paclitaxel.

Next, we asked if AI-driven MN^+^-RBC quantification from WSIs could detect changes in the frequency of spontaneous micronucleus formation associated with inherited genomic instability. To test this, we quantified the frequency of MN^+^-RBCs from mice of multiple genetic backgrounds, including severe combined immunodeficient (SCID) mice carrying inactivating mutations in the DNA repair non-homologous end-joining (NHEJ) factor, *Prkdc*. We observed a frequency of MN^+^-RBCs of 0.139% and 0.090% in gender-matched C57BL/6J and Nude (Rj:NMRI-*Foxn1*^*nu/nu*^) mice respectively, which is in close agreement with previous reports^24^ (Fig. 1E,F). However, we observed that *Prkdc*-deficient SCID mice had a significant 1.9-fold increase in the frequency of MN^+^-RBCs at baseline compared with C57BL/6J mice, consistent with the role of NHEJ in suppressing micronucleus formation^25^ (*P* < 0.0001, Fig. 1E,F). Collectively, these data demonstrate that WSI can be used to quantify the frequency of MN^+^-RBCs to serve either as a PD biomarker following exposure to chemotherapeutic agents or to quantify elevated rates of spontaneous micronucleus formation associated with impairments in the DNA damage response (DDR).

**Fig. 2:**
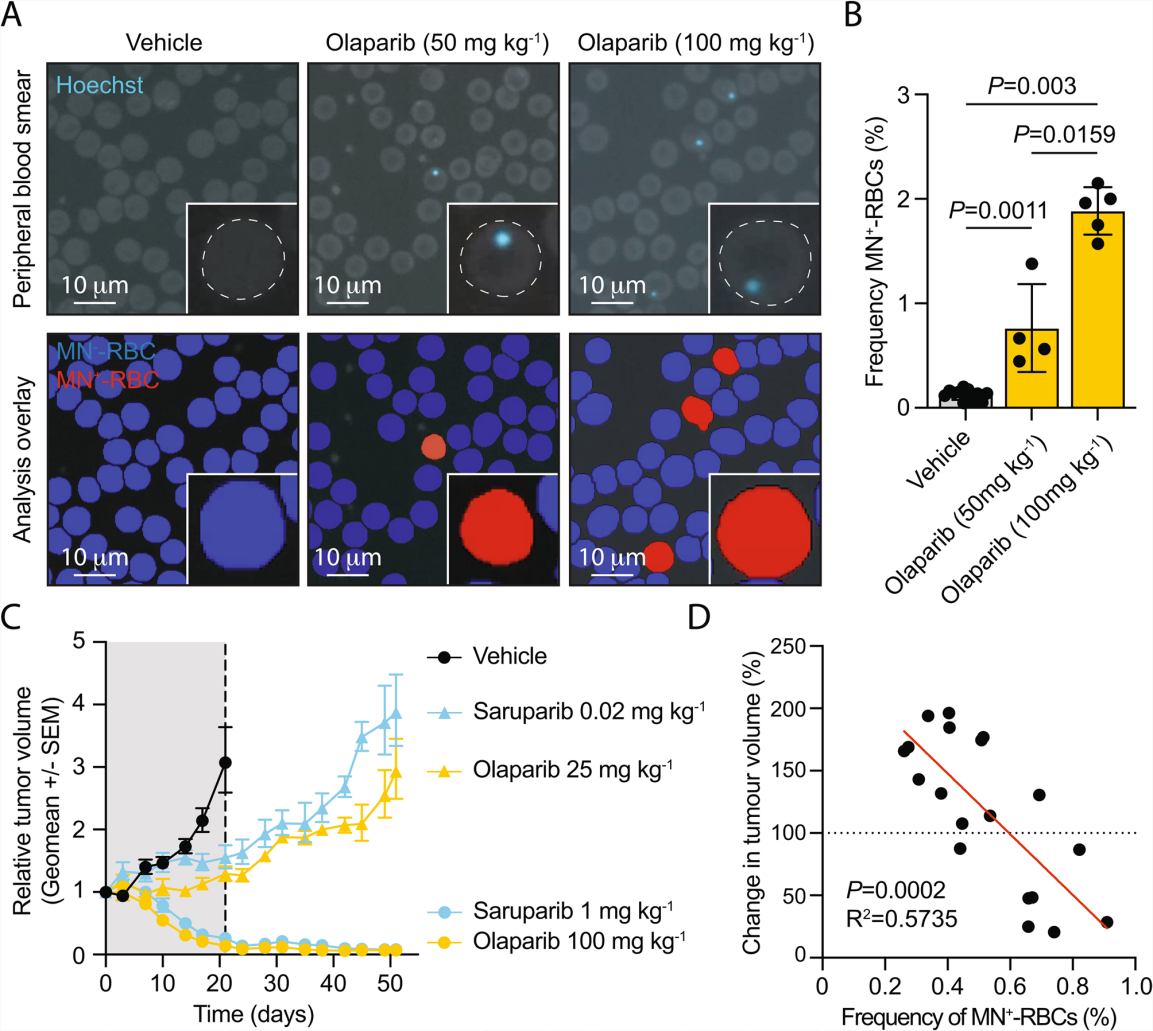
Quantification of micronuclei from whole-slide haematology images provides dose-dependent pharmacodynamic biomarker readouts that correlate with drug efficacy. **A**,**B**, Representative images of Hoechst-stained peripheral blood films with image analysis overlays from C57BL/6J mice exposed to 50 mg kg^-1^ QD or 100 mg kg^-1^ BID olaparib and vehicle-only controls and quantification of the frequency of MN^+^-RBCs (*P*-values calculated using a two-tailed Mann–Whitney *U*-test, data represent the mean ± s.d.; each point represents data from one mouse, *n* = 12, 4 and 5, left to right). **C**, *In vivo* anti-tumor efficacy of olaparib at 25 mg kg^-1^ QD and 100 mg kg^-1^ BID and saruparib at 0.02 mg kg^-1^ QD and 1 mg kg^-1^ QD in the MDA-MB-436 xenograft model (data represent the relative geomean tumor volume and s.e.m.,; starting tumor volume was 0.181-0.349 mm^3^; *n* = 8 vehicle-only, 6 saruparib (0.02 mg kg^-1^), 6 saruparib (1 mg kg^-1^), 3 olaparib (25 mg kg^-1^) and 4 olaparib (100 mg kg^-1^)-treated mice; shaded area represents the dosing period group). **D**, Linear regression analysis of MN^+^-RBC frequency and change in MDA-MB-436 xenograft tumor volume in mice treated for 14 days with either olaparib, saruparib or vehicle-only controls (The data shown represent individual biological replicates, *n* = 19, *P*=0.0002, R^2^=0.5735).

We next asked if MN^+^-RBC quantification from WSIs could serve as a minimally invasive PD biomarker to support pre-clinical drug development. To this end, we first set out to test if MN^+^-RBC quantification from WSIs could provide dose-dependent PD biomarker information following exposure to the clinically approved PARP inhibitor, olaparib. C57BL/6J mice were exposed to either 50 mg kg^-1^ QD or 100 mg kg^-1^ BID of olaparib daily for 3 weeks and peripheral blood smears obtained on the final day of treatment (day 21). We observed significant 6.0- and 14.9-fold increases in the frequency of MN^+^-RBCs following exposure to 50 mg kg^-1^ QD or 100 mg kg^-1^ BID olaparib, respectively (Fig. 2A,B). Next, we asked if the frequency of MN^+^-RBCs correlated with the efficacy of PARP inhibitor therapy in pre-clinical tumor models. To test this, SCID^*Prkdc*^ mice were implanted with the *BRCA1*-deficient, triple-negative breast cancer cell line MDA-MB-436, which harbours the 5396 + 1G>A mutation in the splice donor site of exon 20 in *BRCA1*^26^. This mutation results in aberrant splicing, loss of function of BRCA1 and homologous recombination repair (HRR) deficiency, leading to sensitivity to PARP inhibition. MDA-MB-436 tumor-bearing mice were randomized and exposed to varying doses of either olaparib or the PARP1-selective inhibitor, saruparib^27^. Consistent with previous reports we observed dose-dependent efficacy with both olaparib and saruparib (Fig. 2C)^27,28^. Importantly, we observed a significant correlation between the efficacy of PARPi therapy and the frequency of MN^+^-RBCs following 14 days of exposure (Fig. 2D and Supplementary Figure 3). These data confirm the applicability of MN^+^-RBC analyses from WSIs as a dynamic PD biomarker of drug response, as increasing doses of PARP inhibitors elicit distinct levels of efficacy in pre-clinical tumor models^27^.

**Fig. 3:**
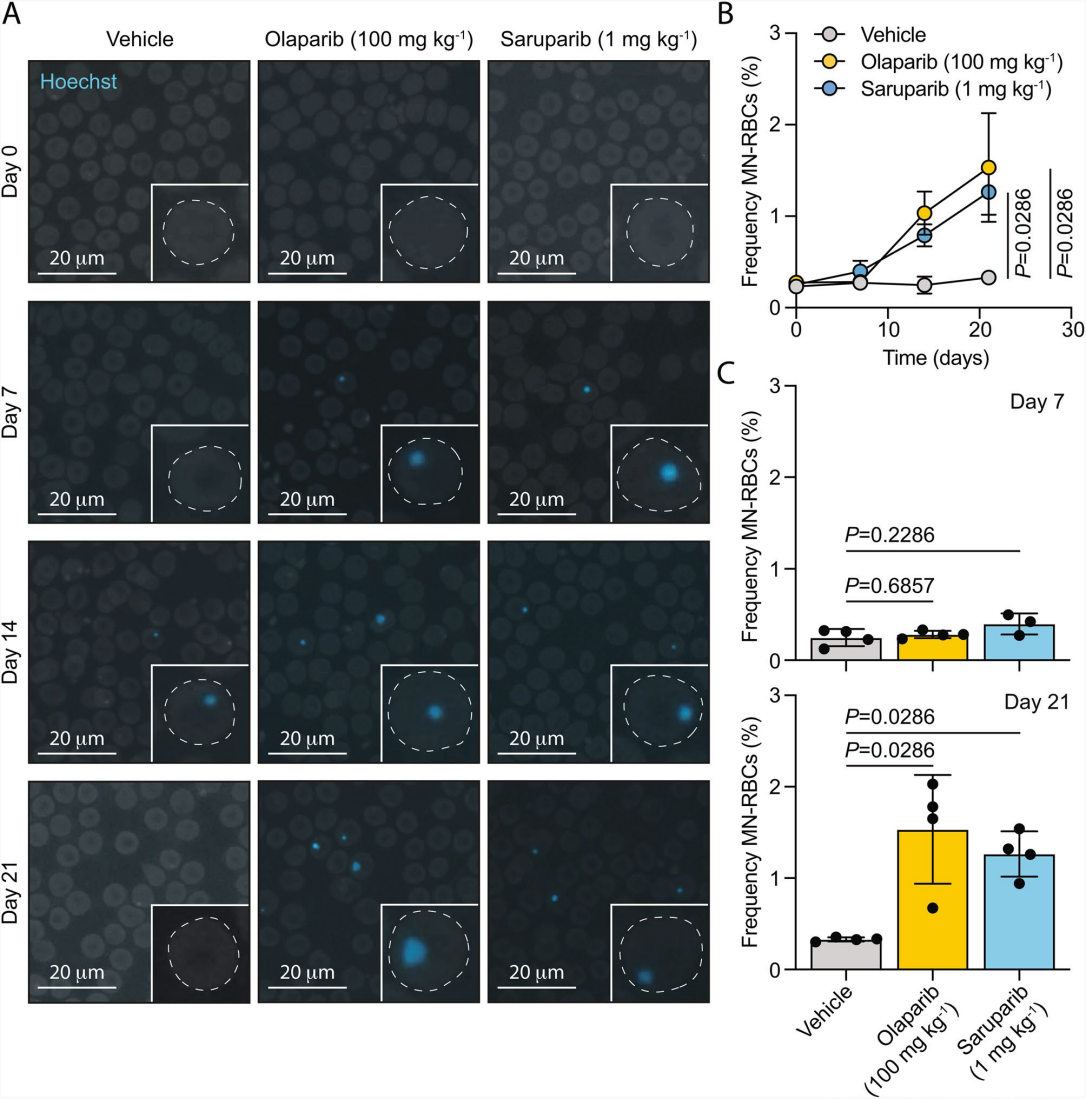
Whole-slide imaging enables longitudinal pharmacodynamic monitoring of PARP inhibitor-induced micronucleus formation. **A**,**B**, Representative images of Hoechst-stained peripheral blood films from SCID^*Prkdc*^ mice exposed to either 100 mg kg^-1^ QD olaparib, 1 mg kg^-1^ QD saruparib or vehicle-only controls and quantification of the frequency of MN^+^-RBCs (*P*-values calculated using a two-tailed Mann–Whitney *U*-test, data are the mean ± s.d.; vehicle, *n* = 5, 2, 4 and 4; olaparib, *n* = 4, 4, 7 and 4; saruparib, *n* = 3, 3, 7 and 4, left to right). **C**, Quantification of the frequency of MN^+^-RBCs from SCID^*Prkdc*^ mice exposed daily to either 100 mg kg^-1^ olaparib, 1 mg kg^-1^ saruparib or vehicle-only controls at day 7 (Top) or 21 (Bottom) days (*P*-values calculated using a two-tailed Mann–Whitney *U*-test, data are the mean ± s.d.; day 7, *n* = 4, 4, and 3 left to right; day 21, *n* = 4, 4 and 4, left to right).

As peripheral blood films can be derived from as little as 5 μl of total blood, we next asked if serial, non-terminal sampling could facilitate longitudinal kinetic profiling of micronucleus induction by WSI. To do this, SCID^*Prkdc*^ mice were exposed to either olaparib (100 mg kg^-1^ QD), saruparib (1 mg kg^-1^ QD) or vehicle-only controls daily and peripheral blood films obtained every 7 days and the frequency of MN^+^-RBCs quantified from WSIs (Fig. 3A,B). Interestingly, we observed no significant increase in the frequency of MN^+^-RBCs after the first 7 days of exposure to either olaparib (*P* = 0.6857) or saruparib (*P* = 0.2286) (Fig. 3C). However, we subsequently observed a time-dependent increase in the frequency of MN^+^-RBCs that culminated in significant 4.7- and 3.9-fold increases following exposure to olaparib or saruparib for 21 days, respectively (Fig. 3B,C).

**Fig. 4:**
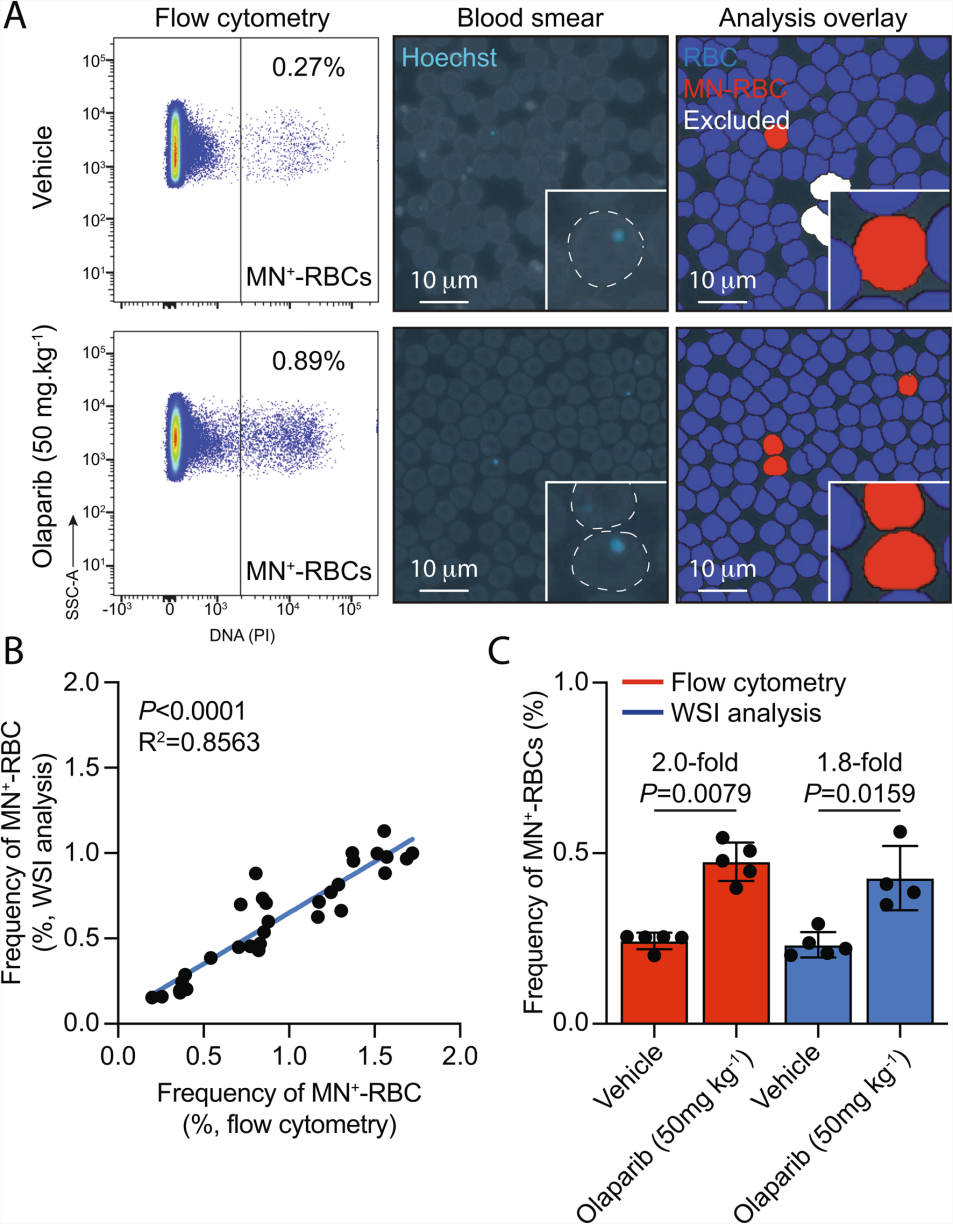
Whole-slide imaging achieves strong analytical concordance with flow cytometry for MN^+^-RBC quantification. **A**, Representative flow cytometry plots and images of Hoechst-stained blood smears with image analysis overlays of blood samples from mice treated with olaparib (50 mg kg^-1^) or vehicle-only controls. **B**, Linear regression analysis of paired haematology slides and methanol-fixed total blood samples analysed by image analysis and flow cytometry, respectively (The data shown represent individual biological replicates, *n* = 31; *P*<0.0001 and R^2^=0.8563, Pearson’s r = 0.926). **C**, Quantification of MN^+^-RBCs from mice treated with olaparib (50 mg kg^-1^) and vehicle-only controls by either flow cytometry or whole-slide image analysis (*P*-values calculated using a two-tailed Mann–Whitney *U*-test, data are the mean ± s.d.; each point represents data from one mouse, *n* = 5, 5, 5 and 4, left to right).

Having demonstrated that WSI enables quantification of both the magnitude and kinetics of micronucleus formation, we next set out to determine the analytical concordance between WSI and flow cytometry, which represents the mainstay approach for MN^+^-RBC enumeration in research laboratories^24,29^. To do this, we derived paired peripheral blood films and methanol-fixed total blood samples from mice exposed to increasing doses of olaparib or vehicle-only controls (Fig. 4A and Supplementary Figure 4). When we compared the frequency of MN^+^-RBCs as determined by WSI and flow cytometry, we observed a strong correlation (Pearson’s r = 0.926) and significant concordance (R^2^ = 0.8563, *P* < 0.0001, Fig. 4B). Furthermore, we observed comparable 2.0- and 1.8-fold increases in the frequency of MN^+^-RBCs following exposure of mice to 50 mg kg^-1^ olaparib when using flow cytometry or WSI, respectively (Fig. 4C). These data demonstrate substantial analytical concordance between WSI- and flow cytometry-based approaches for the quantification of MN^+^-RBC.

**Fig. 5:**
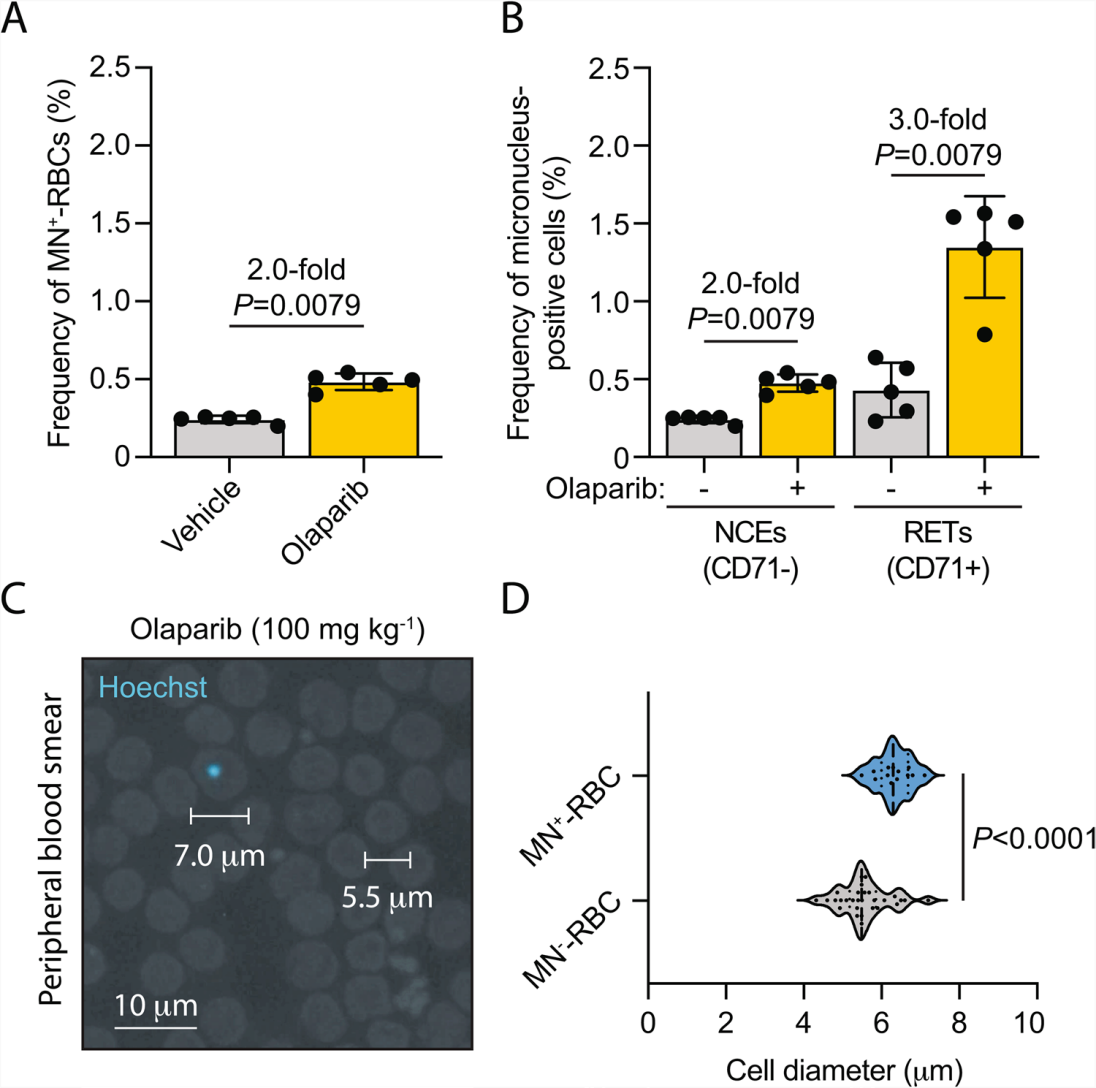
Quantification of micronuclei in reticulocytes provides increased dynamic PD readouts than total RBC analysis. **A**, Quantification of total MN^+^-RBCs (inclusive of both NCEs and RETs) from SCID^*Prkdc*^ mice treated with olaparib (50 mg kg^-1^ QD) and vehicle-only controls by flow cytometry (*P* values calculated using a two-tailed Mann–Whitney *U*-test. The data shown represent the mean and s.d.; each point represents data from one mouse, *n* = 5 mice per group). **B**, Quantification of the frequency of micronucleus-positive normochromic erythrocytes (NCEs, CD71-) and reticulocytes (RETs, CD71+) from SCID^*Prkdc*^ mice exposed to 50 mg kg^-1^ QD olaparib by flow cytometry (*P*-values calculated using a two-tailed Mann–Whitney *U*-test, data are mean ± s.d.; each point represents data from one mouse, *n*=5, 5, 5 and 5, left to right). **C**, Representative image of a Hoechst-stained peripheral blood film from a SCID^*Prkdc*^ mouse treated with olaparib (50 mg kg^-1^ QD). D, Quantification of the diameter of peripheral red blood cells with (MN^+^-RBC) and without (MN^-^-RBC) Hoechst-positive micronucleus structures (*P*-value calculated using a two-tailed Welch’s t-test, data represent the median and interquartile range; *n* = 13 and 29, top to bottom.)

Nevertheless, Hoechst-stained slides do not allow for the sub-classification of RBCs into mature normochromic erythrocytes (NCEs) and immature reticulocytes (RETs), which are readily distinguishable by flow cytometry using an anti-transferrin receptor/CD71 antibody^30^. Importantly, RETs are enriched for micronuclei, which are eventually cleared from circulation as they mature into NCEs. Indeed, exposure of SCID^*Prkdc*^ mice to 50 mg kg^-1^ olaparib daily for 14 continuous days resulted in a significant 2.0-fold increase in the frequency of MN^+^-RBCs (inclusive of both the NCE and RET populations) as determined by flow cytometry (Fig. 5A and Supplementary Figure 4). However, when the total RBC population is stratified into CD71-negative NCEs and CD71-positive RETs, we observed a significant 2-fold increase in the frequency of MN^+^-NCEs, but a greater 3.0-fold increase in the frequency of MN^+^-RETs following exposure to olaparib (Fig. 5B). Therefore, RETs represent the population with the most dynamic changes in micronucleus frequency, information that is not adequately captured by Hoechst staining alone.

Nevertheless, while WSIs of Hoechst-stained smears did not allow for the identification of RETs, we did observe that MN^+^-RBCs were significantly larger than surrounding MN^-^-RBCs. These data are consistent with an enrichment of micronuclei in immature RETs, which are reported to be larger than mature NCEs^31^ (Fig. 5C,D). Therefore, to maximise the utility of WSI-based MN^+^-RBC quantification, we stained haematology slides with May-Grünwald Giemsa (MGG) dyes, a Romanowsky-type stain that allows the concurrent identification of both RETs (which stain with a bluish-grey hue and appear slightly larger than mature NCEs) and micronuclei (small circular structures that stain purple). Importantly, Romanowsky stains, including MGG, represent the most frequently used stains for the analysis of haematology specimens in clinical practice^32^.

**Fig. 6:**
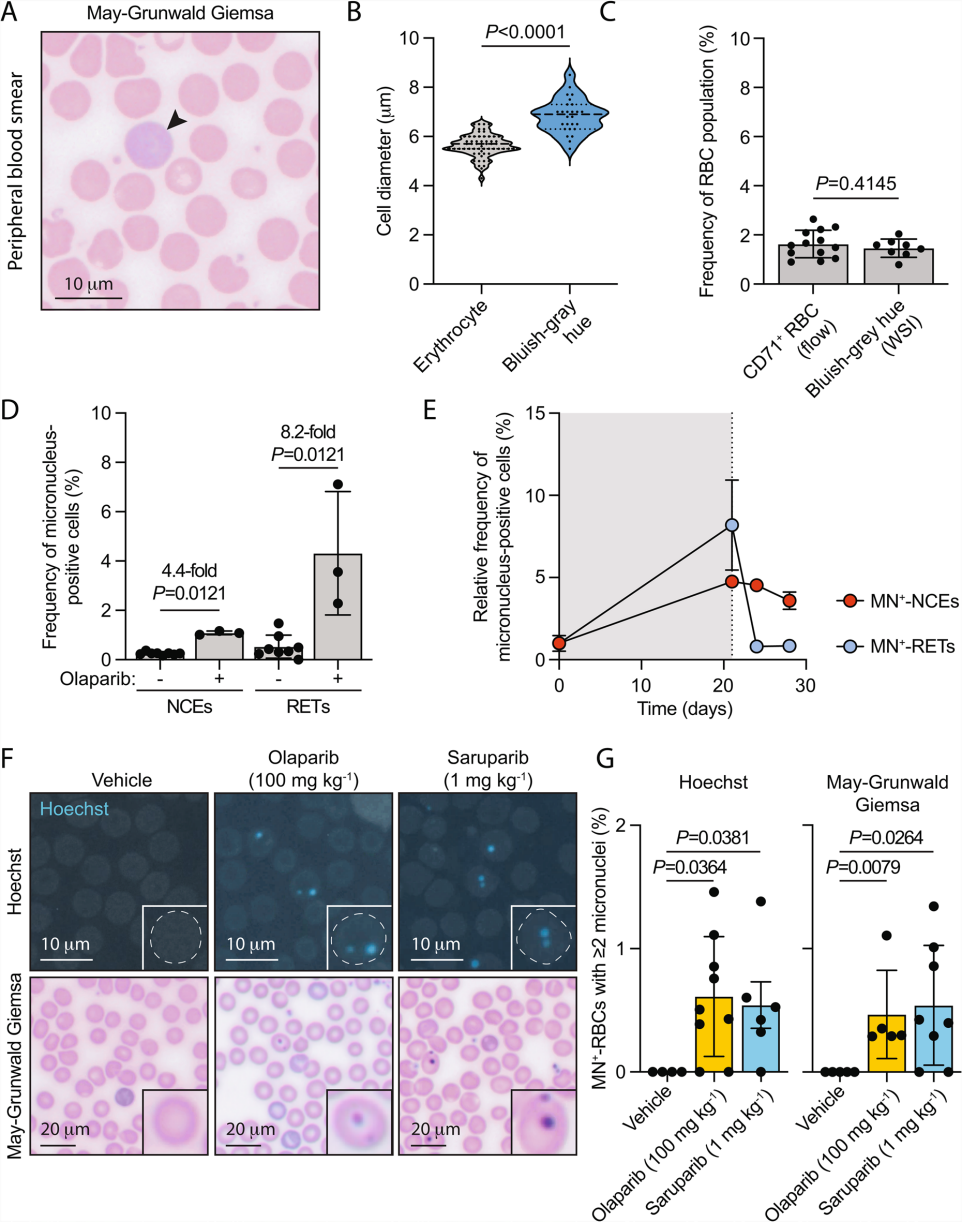
Whole-slide imaging of May-Grünwald Giemsa (MGG)-stained haematology slides facilitates the quantification of micronucleus-positive reticulocytes. **A**, Representative image of MGG-stained peripheral blood film from treatment naive SCID^*Prkdc*^ mice (arrowhead denotes a large Bluish-grey-stained reticulocyte). **B**, Quantification of cell diameter (*P*-value calculated using a two-tailed Welch’s t-test*,* data shown represent the median and interquartile range; *n* = 59 and 26, left to right). **C**, Quantification of the frequency of reticulocytes from SCID^*Prkdc*^ mice as determined by either flow cytometry using an anti-transferrin/CD71 antibody and from whole-slide images of May-Grünwald Giemsa-stained slides (Bluish-grey hue) (*P*-value calculated using a two-tailed Welch’s t-test, data represent the mean ± s.d.; each point represents data from one mouse, *n* = 13 and 8, left to right). **D**, Quantification of the frequency of micronucleus-positive normochromic erythrocytes (NCEs) and reticulocytes (RETs) from SCID^*Prkdc*^ mice exposed to 100 mg kg^-1^ QD olaparib for 21 days (*P*-values calculated using a two-tailed Mann–Whitney *U*-test, data are mean ± s.d.; each point represents data from one mouse, *n*=8, 3, 8 and 3, left to right). **E**, Quantification of the frequency of micronucleus-positive normochromic erythrocytes (NCEs) and reticulocytes (RETs) from SCID^*Prkdc*^ mice exposed to 100 mg kg^-1^ QD olaparib for 21 days and for 7 days post-exposure (*P*-values calculated using a two-tailed Mann–Whitney *U*-test, data are mean ± s.d.; each point represents data from one mouse, NCE, *n*=5, 3, 2 and 4; RET, *n* = 5, 3, 2 and 3, left to right). **F**,**G**, Representative images of peripheral blood smears from mice exposed to either 100 mg kg^-1^ QD olaparib, 1 mg kg^-1^ saruparib QD or vehicle-only controls for 21 days stained with Hoechst or May-Grünwald Giemsa dyes and the quantification of red blood cells containing 2 or more micronuclear structures (*P*-values calculated using a two-tailed Mann–Whitney *U*-test, data are mean ± s.d.; each point represents data from one mouse; Hoechst, *n*=4, 9 and 6; May-Grünwald Giemsa, *n* = 5, 5 and 8, left to right).

To subclassify MN^+^-RBCs into MN^+^-RETs and MN^+^-NCEs from WSIs of MGG-stained blood smears, we developed a supervised deep-learning algorithm using pathologist-guided annotations (Supplementary Figure 5A). Consistent with the identification of RETs, the cells identified by the algorithm as RETs that stained with a bluish-grey hue were significantly larger than adjacent biconcave NCEs and occurred at a frequency of 1.46% of circulating RBCs (Fig. 6A-C). Furthermore, we observed no significant difference in the frequency of RETs identified using our deep-learning algorithm on WSIs compared to flow cytometry methods using a fluorescently labelled antibody against CD71, which is expressed on the surface of RETs (*P* = 0.4145, Fig. 6C).

Having established a method to subclassify MN^+^-RBCs from WSIs, we next asked if restricting micronucleus quantification to RETs provides increased dynamic range. To test this, we stained peripheral blood films from mice exposed to 100 mg kg^-1^ olaparib or vehicle-only controls for 21 days with MGG and quantified both the frequency of MN^+^-NCEs and MN^+^-RETs. Following exposure to olaparib, we observed a significant 4.4-fold increase in the frequency of MN^+^-NCEs and a larger, significant 8.2-fold increase in the frequency of MN^+^-RETs (Fig. 6D). Interestingly, when we mapped the kinetics of micronucleus formation, we observed that following 21 days of exposure to olaparib the relative frequency of both MN^+^-NCEs and MN^+^-RETs was significantly increased (Fig. 6E). However, following treatment cessation, the frequency of MN^+^-RETs declined rapidly, returning to baseline levels within 72 hours, whilst the frequency of MN^+^-NCEs remained stable for 72 hours and declined only modestly after 7 days (Fig. 6E). The rapid reduction in the frequency of circulating MN^+^-RETs after treatment cessation is consistent with both a halt in micronucleus formation in newly produced immature blood cells and the maturation of existing circulating RETs into NCEs. Consistent with this, we observed greater inter-animal variability in the frequency of MN^+^-RETs compared to MN^+^-NCEs (Supplementary Figure 6), which likely reflects the dynamic nature of the reticulocyte population and is consistent with previous reports^30^. Collectively, these data demonstrate that deep learning algorithms can be used to quantify MN^+^-RETs from WSIs of MGG-stained slides, without the need for immunostaining or flow cytometry.

Furthermore, we hypothesised that high-resolution WSIs would facilitate the analysis of additional cellular features potentially over-looked when quantifying micronuclei by flow cytometry. Specifically, we set out to quantify the frequency of RBCs harbouring multiple micronuclei, a phenotype that is disregarded following the binary classification of RBCs as either micronucleus-positive or negative by flow cytometry gating strategies (Supplementary Figure 4). Strikingly, we found a significant increase in the frequency of RBCs with $\ge$2 micronuclei in mice following exposure to either 100 mg kg^-1^ olaparib or 1 mg kg^-1^ saruparib (Fig. 6F,G). To further validate these data, we assessed the frequency of multi-micronucleated RBCs using both fluorescent Hoechst DNA dyes and MGG dyes and observed comparable fold inductions across both staining methods. Collectively, these data demonstrate the advantages of WSI for micronucleus quantification, including the sub-classification of RBCs and the detection of multiple micronuclei.

Finally, we set out to maximise the translatability of WSI-based MN^+^-RBC quantification by defining the impact of pre-analytical factors on peripheral blood smear quality. To do this, we assessed the consequences of prolonged blood storage prior to smear preparation on sample quality by deriving sequential smears from whole blood samples stored either at room temperature (RT) or at 4°C over a 48-hour period (Supplementary Figure 7). Using pathologist-guided annotations we developed a supervised deep learning algorithm to quantify morphological features associated with sample deterioration, including RBC crenation and elongation (Supplementary Figure 5B). Interestingly, we observed no significant difference in the frequency of echinocytes (crenated red blood cells displaying membrane spicules) in samples stored up to 6 hours at 4°C (*P* = 0.1645), however, we did observe a significant 51-fold increase when samples were stored at room temperature for 6 hours (*P* = 0.0280, Supplementary Figure 7A, B). Furthermore, we observed a concomitant increase in the frequency of other morphological abnormalities, including elongated red blood cells (Supplementary Figure 7A,C). Nevertheless, despite the degeneration of sample quality following prolonged storage, we observed no significant change in the frequency of reticulocytes following storage up to 24 hours (Supplementary Figure 7D). Collectively, these data demonstrate the importance of sample stewardship and appropriate pre-analytical considerations, whilst also highlighting the advantage of WSI-based approaches to micronucleus quantification that enable the integration of algorithmic quality control assessment of sample suitability prior to downstream analysis.

## Discussion

Micronuclei are sub-cellular compartments that sequester entire chromosomes or chromosomal fragments that become isolated following an aberrant mitotic division, often caused by genomic insults that result in lagging chromosomes, DNA breaks or anaphase bridges^33^. Their formation is a hallmark of chromosomal instability and DNA damage, both in tumor cells and normal bone marrow progenitors^1^. Consequently, micronucleus quantification is a well-established pharmacodynamic biomarker for anti-cancer therapies, yet traditional quantification methods are labour-intensive, low-throughput and not readily deployable in the clinic. Furthermore, the acquisition of tumor material from patients is invasive, and the faithful identification of micronuclei from routine histopathology slides has hindered the broad adoption of micronucleus-based biomarker assessments.

Fortuitously, the haematopoietic system offers a readily accessible surrogate system to monitor micronucleus formation *in vivo*. During erythropoiesis, developing red blood cells expel their nucleus, giving rise to immature enucleated red cells that enter peripheral circulation^34^. Importantly, micronuclei are frequently retained within developing reticulocytes following nuclear expulsion, and in murine models these nuclear remnants are retained during reticulocyte maturation and are readily detected within mature normochromic erythrocytes^24,35^.

Consequently, automated imaging-based micronucleus scoring has evolved from plate-based fluorescent image analysis for *in vivo* rodent assays, to deep learning approaches achieving near-human performance (r > 0.95) in culture cell lines to quantify chromosomal instability^36–38^. However, the clinical translatability of these methods has been limited by reliance on *in vitro* cell culture or the use of complex fluorescent dyes requiring specialised imaging systems. However, the rapid emergence of whole-slide imaging and advances in artificial intelligence in digital pathology now enable the automated quantification of micronuclei in peripheral blood using standard histological stains at scale, creating opportunities for translatable pharmacodynamic biomarker assay development.

Here, we couple whole-slide imaging technologies and supervised deep learning algorithms to quantify micronucleated red blood cells from routine haematology slides. We report strong analytical concordance between whole-slide imaging and established flow cytometry methods for the quantification of micronucleus-positive red blood cells (MN^+^-RBCs). We observed significant concordance (Fig. 4B; Pearson’ r = 0.925, *P* < 0.0001) between the two approaches, with both methods yielding comparable fold-increases in MN^+^-RBC frequency following treatment with 50 mg kg^-1^ olaparib (2.0-fold and 1.8-fold for flow cytometry and WSI, respectively). These results provide compelling evidence that WSI-based quantification is equivalent to gold-standard techniques like flow cytometry. This validation supports the utility of whole-slide images of haematology specimens as a reliable and scalable alternative for MN^+^-RBC scoring.

A major advantage of the AI-driven approaches described here is the ability to stratify red blood cells into mature erythrocytes and immature reticulocytes using only standard haematology stains, such as the May-Grünwald Giemsa dyes, without the need for immunostaining. Computational pathology approaches, such as the supervised deep-learning approaches described here, leverage subtle morphological features and staining patterns to distinguish cell populations, allowing the simultaneous identification and quantification of micronuclei within reticulocytes. Additional advantages of whole-slide imaging approaches are the potential for high throughput screening, where many slides can be stained, scanned and analysed in a semi-automated fashion, enabling high-throughput workflows that are considerably less labour-intensive than manual scoring using a brightfield microscope, and require less sample processing than flow cytometry, which requires the immediate processing of fresh haematological specimens. These advantages underscore the potential of digital pathology workflows to enhance both the efficiency and robustness of MN^+^-RBC quantification as a clinically deployable pharmacodynamic biomarker.

Indeed, we provide evidence that the quantification of micronuclei within peripheral red blood cells demonstrates promising utility for monitoring drug-induced pharmacodynamic changes *in vivo*. Our findings suggest that this approach provides a sensitive, minimally invasive readout of pharmacological activity of chemotherapeutic agents and clinically relevant PARP inhibitors. However, it is important to recognise that the quantification of micronuclei in peripheral erythrocytes reflects a biological response in non-tumor cells, which may not fully recapitulate tumor-specific responses. The uncoupling of tissue-specific responses represents a potential limitation when extrapolating pharmacodynamic biomarker changes in peripheral blood cells to tumor-specific PD and efficacy.

Our findings have important implications for pre-clinical drug development. The minimal blood volume requirement (5 μl) of WSI-based MN^+^-RBC quantification enables serial, minimally invasive sampling for longitudinal pharmacodynamic monitoring in individual subjects, addressing a current limitation in early oncology research. The demonstrated correlation between MN^+^-RBC induction and anti-tumor efficacy (Figure 2D) suggests that this biomarker could serve as an early indicator of therapeutic activity, potentially enabling dose optimisation and candidate selection in early drug discovery. For DNA-damaging agents and inhibitors of the DNA damage response, establishing pharmacodynamic activity in readily accessible peripheral blood samples could accelerate preclinical development by providing rapid feedback on target engagement and biological activity. Beyond pre-clinical applications, this approach has potential to enhance clinical trial design. In early-phase dose-escalation trials, serial MN^+^-RBC monitoring could provide objective pharmacodynamic evidence of biological activity. Furthermore, in comparison to tumor biopsy-based biomarkers, MN^+^-RBC quantification offers several practical advantages, including minimally invasive sampling that can be performed more frequently and provides a systemic readout that may better reflect overall pharmacodynamic profiles than a singular biopsy that may not fully represent disease heterogeneity.

## Limitations and future work

Several important limitations should be considered when interpreting our findings, and these highlight key areas for future investigation. Firstly, our experiments were conducted exclusively in mouse models, and direct extrapolation to humans requires careful validation. While the fundamental biology of erythropoiesis and micronucleus formation is conserved between mice and humans, important species-specific differences exist. Notably, mature murine erythrocytes retain micronuclei in peripheral circulation, whereas in humans these structures are typically removed by splenic filtration^39,40^. Consequently, baseline frequencies and kinetics of MN^+^-RBCs formation may differ between species. Secondly, our xenograft efficacy studies utilised immunocompromised SCID^*Prkdc*^ mice, which exhibit elevated baseline micronucleus frequencies, likely due to NHEJ deficiency (Figure 1E, F)^25^. While this model is standard for evaluating anti-tumor efficacy, the relationship between MN^+^-RBC induction and therapeutic response may differ in immunocompetent mice, or in patients with intact DNA repair pathways. Third, we acknowledge that micronucleus quantification in peripheral blood cells represents a surrogate biomarker that may not fully recapitulate tumor-specific responses. The uncoupling of tissue-specific responses represents a potential limitation when extrapolating pharmacodynamic changes in peripheral circulation to tumor-specific effects. Nevertheless, the correlation observed between MN^+^-RBC induction and tumor growth inhibition suggests that despite tissue-specific differences, peripheral blood responses can reflect therapeutically relevant drug activity (Figure 2D). Finally, we did not systematically evaluate potential confounding factors that might influence MN^+^-RBC frequencies in clinical populations, including age, concurrent medications, comorbidities or prior treatment exposures^24,41^. Despite these limitations, our study provides proof-of-concept that automated WSI-based MN^+^-RBC quantification offers a scalable, minimally invasive approach for pharmacodynamic biomarker monitoring.

## Conclusion

In summary, the computational pathology workflow described here provides a robust and scalable approach for the quantification of micronuclei in red blood cells as a surrogate pharmacodynamic biomarker, achieving analytical concordance with flow cytometry (r = 0.926) from only 5 μl of blood. While further validation in diverse clinical settings is required, these findings provide proof-of-concept that computational pathology workflows may facilitate scalable, automated biomarker quantification for pharmacodynamic assessment in translational research settings.

## Methods

### Mice

All animal experimental protocols were conducted in accordance with UK Home Office legislation, the Animal Scientific Procedures Act 1986, and the AstraZeneca Global Bioethics policy (https://www.astrazeneca.com/content/dam/az/Sustainability/Bioethics_Policy.pdf) and in accordance with PREPARE and ARRIVE guidelines. All experimental protocols are outlined in the project licence PP3292652, which was approved by ethical review at AWERB, an ethical review body that ensures animal welfare and promotes the 3Rs (Replacement, Reduction, Refinement) and approved by the UK Home Office. All mice were housed in autoclaved cages with access to food and water *ad libitum* in a sterile environment maintained with a 12 h dark/12 h light cycle at 72 ± 2 °F with 50 ± 20% room humidity. All mouse studies were carried out using female mice. C57BL/6 and SCID^*prkdc*^ female mice were obtained from Envigo. Nude (Rj:NMRI-*Foxn1*^*nu/nu*^) mice were obtained from Charles River UK. All mice were received from commercial suppliers at 5-to-7-weeks of age and were allowed to acclimate for a minimum of 7 days before any experimental procedures. All experiments were initiated when mice were 6-to-8-weeks of age. At the completion of experiments, mice were humanely euthanized via cervical dislocation immediately followed by exsanguination via severing of a major artery/permanent cessation of circulation.

For acute exposure to cisplatin, unless otherwise stated 6-to-8-week-old mice were administered cisplatin at 4 mg kg^-1^ via intraperitoneal injection (IP) at 10 ml kg^-1^ once per week. For acute exposure to paclitaxel, unless otherwise stated 6-to-8-week-old mice were administered paclitaxel at 20 mg kg^-1^ via intraperitoneal injection (IP) at 10 ml kg^-1^ once per week. For exposure to olaparib or saruparib, mice were dosed via oral gavage either daily (QD) or twice daily (BID) at the indicated dose in a volume of 10 ml kg^-1^, as described previously^42^. Saruparib was formulated in hydrochloric acid (0.5%) in distilled water adjusted to pH 3.4-4^27^, while olaparib was formulated in 10% DMSO, 50% of 60% Kleptose and 40% water.

For xenografts studies MDA-MB-436 cells were cultured as described in Supplementary Table S1 and cell viability assessed using an automated EVE cell counter (NanoEnTek). Xenografts were established by subcutaneous injection of 2 x 10^6^ viable MDA-MB-436 cells suspended in 50% Matrigel (Corning) in RPMI into the left flank of 6-to-8-week-old SCID^*Prkdc*^ mice under isoflurane anaesthesia (Vet-Tech dispenser) at 0.1 ml per animal, with microchipping performed at the same time for animal identification. All animals were implanted simultaneously with the same MDA-MB-436 suspension and tumors were allowed to establish for 34 days to reach tumor volumes of approximately 0.1 – 0.3 cm^3^. After 34 days, 76% of animals implanted reached appropriate tumor volumes (range 0.181 to 0.349 cm^3^) before randomization into treatment groups to ensure balanced starting tumor sizes (Supplementary Figures 8), as described previously^27,28^. Tumors were measured by caliper and the tumor volume calculated using the following formula: [length (mm) x width (mm)^2^] x π/6 where the length and the width are the longest and the shortest diameters of the tumor respectively (Supplementary Table S2). Following randomisation animals were weighed daily throughout the study (Supplementary Figure 9**).**

Relative tumor volume (RTV) was calculated using the formula:

$${RTV}_{Day X}= \frac{{ TV}_{Day X }}{{ TV}_{Day 0}}$$

### Blood collection and peripheral blood film production

Total blood was obtained either via tail bleed or cardiac puncture into K_3_EDTA MiniCollect tubes and processed immediately, unless otherwise stated in the text (Supplementary Table S3). For peripheral blood films, 5 μl of total blood was placed onto a Superfrost Plus microscope slide (Sigma) aboard a CellVision HemaPrep® (Sysmex) semi-automated blood smearing device and smeared following the manufacturer’s instructions. Freshly prepared peripheral blood films were left to air dry for 24 hours and stored at room temperature in the presence of desiccants until subsequent analysis. For micronucleus quantification by flow cytometery, 62 μl of total blood was diluted in 338 μl solution of heparin in PBS (500 U ml^-1^, Calbiochem) and 360 μl of the heparin blood suspension was diluted in 3.6 ml methanol chilled to −80°C and stored at −80°C until subsequent analysis.

### Preparation of mouse bone marrow smears

Treated or untreated mice were culled by cervical dislocation and femurs were harvested and placed into pre-chilled PBS. Bone marrow cells were flushed with 2 ml of ice-cold PBS and filtered through a 70 μm cell strainer. Cell suspensions were centrifuged at 1,100xg for 10 minutes and resuspended in 200 μl of PBS before being placed onto glass slides, smeared and left to air dry overnight. Subsequently, air-dried samples were fixed in methanol pre-chilled to -20°C for 5 minutes and allowed to fully dry. Fixed slides were washed once with TBS supplemented with 0.05% w/v Tween20 (Severn Biotech Ltd, 20-7310-10) for 1 minute and stained with haematoxylin and eosin (H&E Stain System S1, 3801654, Leica) using the HistoCore (HistoCore SPECTRA CV X1, Leica) and coverslips (catalog no 3800152, Leica) placed onto slides using a HistoCore SPECTRA workstation (Leica).Whole-slide images were acquired using an Aperio GT450 scanner (Leica) and visualised using eSlideManager software (Leica BioSystems).

### Micronucleus quantification by flow cytometry

The micronucleus assay was performed as described previously^30,43,44^. Briefly, treated or untreated mice were bled and 360 μl of heparin blood suspension was diluted in 3.6 ml pre-chilled methanol and stored at -80°C until subsequently analysis. 1ml of methanol fixed blood was subsequently washed 3 times each for 5 min in 6 ml bicarbonate buffer (0.9% (w/v) NaCl, 5.3 mM NaHCO_3_) at 4°C and resuspended in 50 μl bicarbonate buffer. 20 μl of blood suspension was stained with 80 μl bicarbonate buffer supplemented with 5 μg ml^-1^ FITC-conjugated anti-CD71 antibody (catalog no. 11-0711-82, ThermoFisher Scientific) and 1,000 units.ml^-1^ RNase A (catalog no. R4642, Sigma-Aldrich) and incubated on ice for 45 minutes. Stained blood was washed and resuspended in 500 ul bicarbonate buffer supplemented with 5 μg ml^-1^ propidium iodide (Sigma). Samples were analysed immediately on an LSRFortessa (BD) analyser, with a PBS wash step in between each specimen, for forward scatter (FSC), side scatter (SCC), FITC and PI and the data analysed using FlowJo v.10.10.0. FSC and SSC profiles were used to identify intact blood cells and exclude debris.

### Micronucleus quantification from whole-slide images by image analysis

For micronucleus quantification using Hoechst dyes, air-dried peripheral blood films were fixed in methanol pre-chilled to -20°C for 5 minutes and left to air dry. Fixed slides were washed once with TBS supplemented with 0.05% w/v Tween20 (Severn Biotech Ltd, 20-7310-10) for 1 minute and stained with TBS 0.05% w/v Tween20 supplemented with 8 μg ml^-1^ Hoechst 33342 (catalog no. H3570; Thermo Fisher Scientific) for 5 minutes at room temperature. Slides were mounted using ProLong Diamond Antifade Mountant (catalog no. P36961; Thermo Fisher Scientific) and coverslips (catalog no. 3800145ACS, Leica) placed onto slides. Whole-slide images were acquired at 40x magnification using a Vectra Polaris^TM^ (Akoya Biosciences) slide scanner and images analysed using Visiopharm Software (v2024.07; Visiopharm A/S). For deep learning algorithm development using Hoechst-stained peripheral blood samples, a U-Net convolutional neural network (CNN) architecture was implemented in Visiopharm Software (v2024.07). Training was performed on pathologist-guided annotations where an expert operator classified individual cells as either; micronucleus-positive RBCs (MN^+^-RBCs), micronucleus-negative RBCs (MN^-^-RBCs), white blood cells (WBCs), platelets, and debris. Annotations and training were performed across multiple whole-slide images representative of diverse treatment conditions (vehicle-only controls, olaparib-treated, cisplatin-treated and paclitaxel-treated) and different mouse strains (C57BL/6J and SCID^*Prkdc*^) to ensure model generalisation across experimental contexts. For analysis, regions of interest were manually selected, and the CNN-based algorithm was employed to segment and classify cells. Following initial cell segmentation and classification, a series of post-processing steps were applied to refine cell identification and exclude artifacts. First, WBC and platelet masks were excluded from downstream processing. Second, juxtaposed cells were separated by applying mask erosion (3 pixels), object separation (assuming elliptical geometry with an anticipated diameter of 4 μm), and mask re-dilation (3 pixels). Third, cellular aggregates and debris were excluded using size exclusion thresholds (<7 mm^2^ and >50 mm^2^, respectively), as shown in Supplementary Figure 1A. Following post-processing steps, the frequency of micronucleus-positive RBCs was calculated as:

$$MN+RBCs (\%)= \frac{Number of MN+RBCs}{(Number of MN-RBCs)+(Number of MN+RBCs)}$$

To establish a minimum cell count threshold, we performed subsampling analysis on olaparib-treated samples, demonstrating that the coefficients of variation decreased from 160% when <100 cells were analysed to 3.7% when ≥10,000 cells analysed (Supplementary Figure 1B). Samples with fewer than 10,000 evaluable RBCs were excluded from analysis and not reported.

For micronucleus quantification using May-Grünwald Giemsa dyes, air-dried peripheral blood films were fixed in methanol pre-chilled to -20$^\circ$C for 5 minutes and left to air dry. Fixed slides were washed once in water for 1 minute and stained in May-Grünwald solution (catalog no. ab150670; Abcam) for 10 minutes and washed in phosphate buffer pH 6.8. Slides were then stained in Giemsa solution (catalog no. ab150670; Abcam) for 30 minutes, washed in phosphate buffer, pH 6.8 and air dried at room temperature. Slides were dehydrated in xylene for 5 minutes and mounted with xylene-based mounting media (HistoCore SPECTRA CV X1, Leica) and coverslips (catalog no. 3800152, Leica) placed onto slides using a HistoCore SPECTRA workstation (Leica). Whole-slide images were acquired using brightfield settings at 40x magnification using a Vectra Polaris^TM^ (Akoya Biosciences). Images were analysed using HALO v3.4.4134.309 and HALO-AI v.3.6.4134 (Indica Labs). The image analysis pipeline consisted of two sequential stages. Firstly, cell segmentation was performed using a pre-trained CNN-based segmentation algorithm (HALO-AI v.3.6.4134, Indica Labs) that was further trained using pathologist-guided annotations to identify cell boundaries. Training annotations included cells from diverse regions of blood smears and were performed across multiple whole-slide images representative of diverse treatment conditions to ensure model generalisation across experimental contexts, as described previously. The segmentation algorithm generated individual cell masks for each identified NCE, RET and WBC (Supplementary Figure 5A). Post-processing steps included morphological filtering and size-based exclusion thresholds (<7 mm^2^ and >50 mm^2^) that excluded small debris and cellular aggregates, respectively. Secondly, cell classification was performed using a supervised AI phenotype classifier applied to the cell segmentation masks. The classifier was trained using expert-guided annotations to classify each cell into five categories; micronucleus-negative normochromic erythrocytes (MN^-^-NCEs), micronucleus-positive normochromic erythrocytes (MN^+^-NCEs), white blood cells (WBCs), micronucleus-negative reticulocytes (MN^-^-RETs) and micronucleus-positive reticulocytes (MN^+^-RETs). Following classification, raw cell counts were computed and the frequency of cell populations exported as a percentage of the respective red blood cell population, excluding white blood cells. For each sample, the frequency of micronucleus-positive normochromic erythrocytes (MN^+^-NCEs) was calculated as:

$$MN+NCEs (\%)= \frac{Number of MN+NCEs}{(Number of MN-NCEs)+(Number of MN+NCEs)}$$

Similarly, the frequency of micronucleus-positive reticulocytes (MN^+^-RETs) was calculated as:

$$MN+RETs (\%)= \frac{Number of MN+RETs}{(Number of MN-RETs)+(Number of MN+RETs)}$$

For studies assessing the impact of pre-analytical conditions on peripheral blood films using May-Grünwald Giemsa dyes, an additional classifier was trained to identify morphological abnormalities associated with sample degradation. Using the same segmentation workflow (HALO-AI v.3.6.4134, Indica Labs), cell classification was performed using a supervised AI phenotype classifier trained with pathologist-guided annotations to classify cells into five categories; normochromic erythrocytes (NCEs), white blood cells (WBCs), reticulocytes (RETs), echinocytes (crenated cells displaying characteristic membrane spicules) and elongated RBCs (cells with abnormally elongated morphology, aspect ratio approximately >2.5) (Supplementary Figure 5B). Raw cell counts were computed and the frequency of morphological abnormalities exported as a percentage of the total RBC population. Samples with inadequate smear quality or technical issues during slide preparation (e.g., slide breakage) were excluded from whole-slide image analysis.

### Statistical analysis

Data are shown as mean $\pm$standard deviation (s.d.) unless otherwise stated, and number of independent biological and technical replicates (*n*) are defined in the figure legends. For data distributions presented as violin plots both interquartile ranges and median values are indicated with individual data points overlaid. Normality of quantitative data was assessed using the Shapiro-Wilk test and the two-tailed Welch’s t-test employed for data meeting the normality assumption, while the non-parametric Mann-Whitney *U*-test was employed for data that did not meet the normality assumption. Coefficients of variance (CV) were calculated as the ratio of the standard deviation to the mean, expressed as a percentage. Linear relationships between continuous variables were assessed using linear regression analysis, R^2^ values and associated *P*-values are reported for all regression analyses. For multiple group comparisons the non-parametric Kruskal-Wallis test was employed for data that did not meet normality assumptions. For method comparison analysis was assessed using Pearson’s correlation coefficients (two-tailed) and linear regression analysis on paired samples. All statistical analysis was performed using Prism v10.1.1 (GraphPad software) and the level of significance was set at *P*<0.05 for all analyses.

## Supplementary Information

Supplementary Information 1.

Supplementary Information 2.

Supplementary Information 3.

Supplementary Information 4.

Supplementary Information 5.

Supplementary Information 6.

Supplementary Information 7.

Supplementary Information 8.

Supplementary Information 9.

Supplementary Information 10.

Supplementary Information 11.

Supplementary Information 12.

Supplementary Information 13.

## Data Availability

The data that support the findings of this study are not openly available and are available from the corresponding author upon reasonable request.
