# Supplementary material for: Micronucleus quantification from whole-slide haematology images using AI serves as a translatable pharmacodynamic biomarker for DNA damage response inhibitors
Source: Sci Rep. 2026 Feb 28;16:11437. doi: 10.1038/s41598-026-41458-7 (PMC13057031; doi:10.1038/s41598-026-41458-7)
Supplement: Supplementary file 13 — Supplementary Information 13. [file 41598_2026_41458_MOESM13_ESM.pdf]

Supplementary Materials

Micronucleus quantification from whole-slide haematology images using AI serves as a translatable pharmacodynamic biomarker for DNA damage response inhibitors

Killian H. R. Yong[1]*, Weronika S. Robak[1]*, Lee Mulderrig[2]*, Adina Hughes[2], Richard Bystry[1], Tanya Wantenaar[1], Gemma N. Jones[1], Maria Udriste[1], Jack Robertson[1], Josep V. Forment[2], Lenka Oplustil O'connor[3] & Ross J. Hill[4]

[1]Translational Pathology, Cancer Biomarker Development, Oncology R&D, AstraZeneca, Cambridge, UK. [2]Bioscience, Oncology Targeted Discovery, Oncology R&D, AstraZeneca, Cambridge, UK. [3]Translational Medicine, Oncology R&D, AstraZeneca, Cambridge, UK. [4]Oncology Global Diagnostics, Oncology Business Unit, AstraZeneca, Cambridge, UK.

*Contributed equally to this work
e-mail: lenka.oplustiloconnor@astrazeneca.com and ross.hill@astrazeneca.com

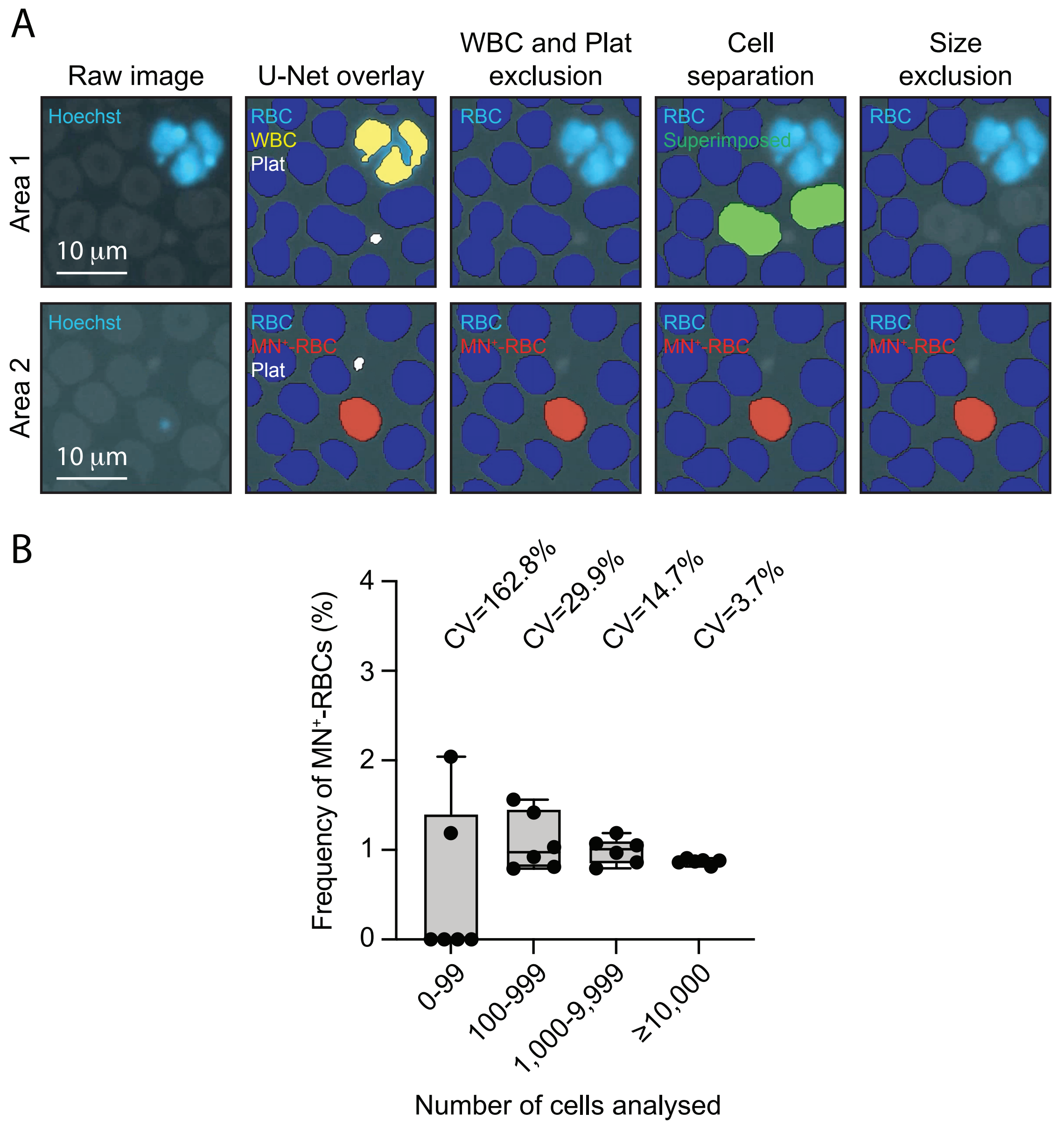

Supplementary Figure 1: Quantifying MN⁺-RBCs from whole-slide images using supervised U-Net convolutional neural networks and intra-animal reproducibility from 5 μl of blood. A, Representative images of Hoechst-stained peripheral blood films from untreated SCID^{Prkdc} mice with algorithmic overlays. Area 1, blue mask, micronucleus-negative red blood cell (RBC); yellow mask, white blood cell (WBC); white mask, platelet (Plat), and green mask, superimposed red blood cells. Area 2, blue mask, micronucleus-negative red blood cell (RBC); red mask, micronucleus-positive red blood cell (MN⁺-RBC); white mask, platelet (Plat). Post-processing steps: exclusion of white blood cells and platelets from analysis pipeline, separation of juxtaposed cells and exclusion of superimposed RBCs as determined by mask area thresholds. **B**, Subsampling analysis of whole-slide images of peripheral blood smears from mice treated with 100 mg kg^{-1} QD olaparib for 14 continuous days showing coefficient of variation (CV) (data are presented as box-and-whisker plots showing median and interquartile range; each point represents data from a randomly selected region of interest, $n = 6$ regions per bin).

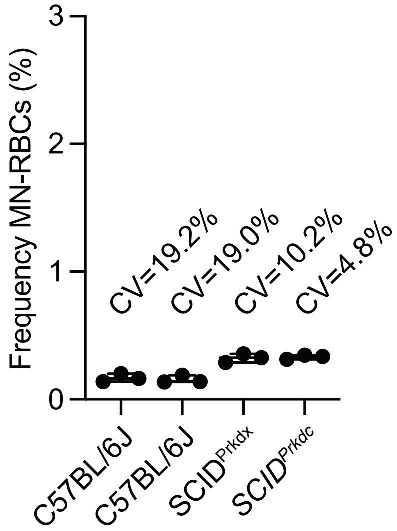

Supplementary Figure 2: Technical reproducibility from 5 μl blood volumes. Quantification of the frequency of MN$^+$-RBCs from multiple independent samples taken from treatment-naïve C57BL/6J and SCID^{Prkdc} mice to assess technical reproducibility. Three technical replicates were obtained from each animal and separate peripheral blood smears were prepared and analysed independently (data are presented as box-and-whisker plots showing median and interquartile range; each point represents data from an independent blood sample per mouse, n = 3 samples per animal, 2 animals per strain; CV, coefficient of variation).

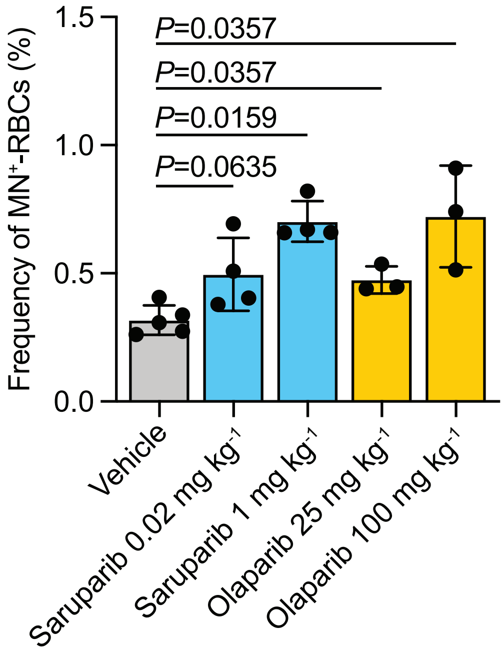

Supplementary Figure 3: Quantification of micronucleus-positive red blood cells in MDA-MB-436 tumor-bearing mice following exposure to PARP inhibitors. Quantification of the frequency of MN^{+}-RBCs from Hoechst-stained peripheral blood smears from MDA-MB-436 tumour-bearing SCID*Prkdc* mice exposed daily to either 0.02 mg kg^{-1} saruparib, 1 mg kg^{-1} saruparib, 25 mg kg^{-1} olaparib, 100 mg kg^{-1} olaparib or vehicle-only controls at day 14 (P-values calculated using a two-tailed Mann–Whitney U-test, data represent the mean ± s.d.; n = 5, 4, 4, 3 and 3, left to right).

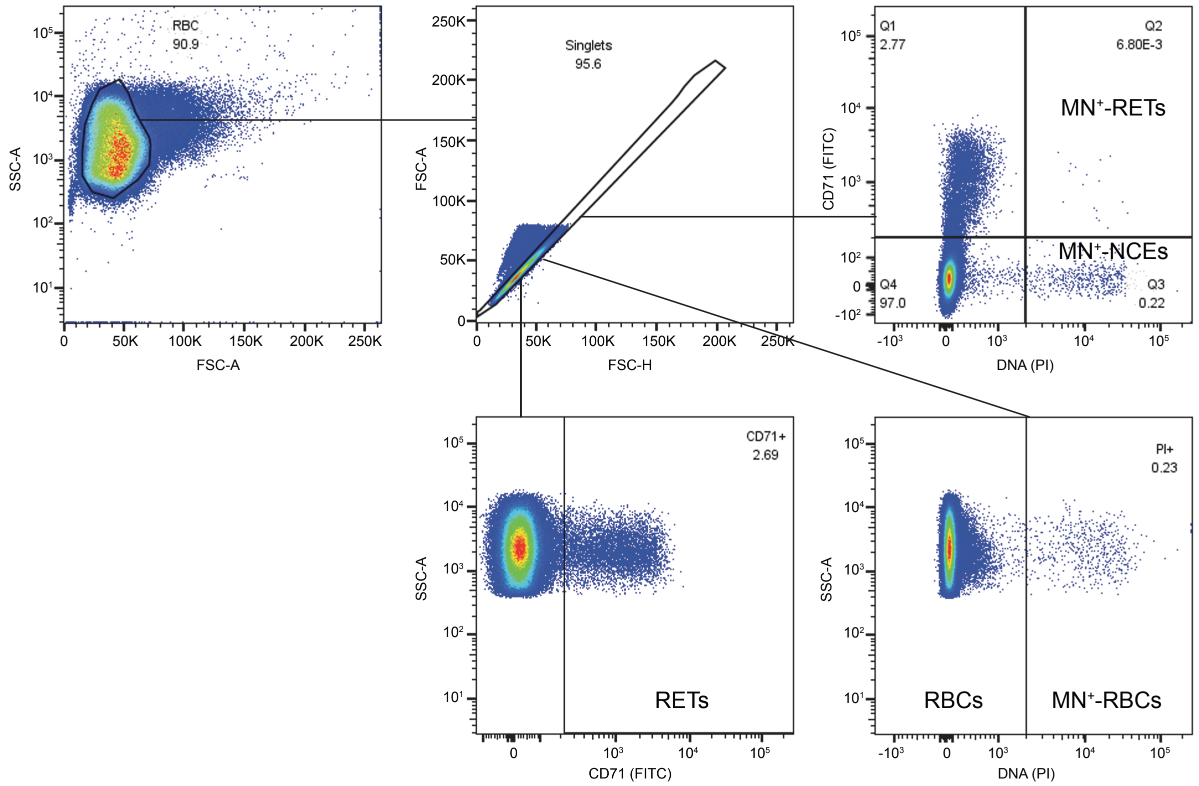

Supplementary figure 4: Flow cytometry gating strategy for micronucleus quantification in red blood cell populations. Sequential gating strategy to quantify the frequency of micronucleated red blood cells (MN+-RBCs, inclusive of both normochromic erythrocytes and reticulocytes), micronucleated normochromic erythrocytes (MN+-NCEs) and micronucleated reticulocytes (MN+-RETs) from methanol-fixed total blood samples. Forward scatter (FSC) and side scatter (SSC) profiles identify intact red blood cells and doublet discrimination is performed using FSC-Area versus FSC-Height to ensure single cell analysis. CD71-FITC expression distinguishes immature reticulocytes (CD71-positive) from mature normochromic erythrocytes (CD71-negative). Propidium iodide (PI) fluorescence identifies micronucleus-positive cells.

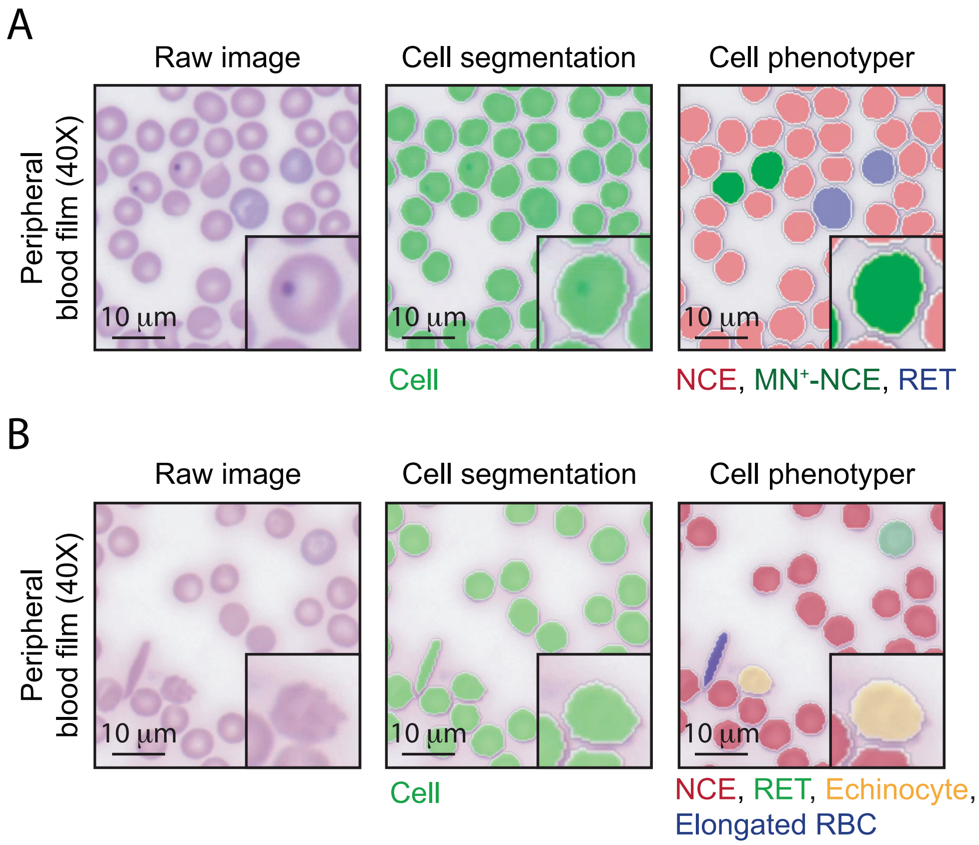

Supplementary figure 5: Quantifying MN$^+$-NCEs, MN$^+$-RETs and morphological defects associated with poor pre-analytical conditions from whole-slide images using supervised deep learning. **A**, Representative image of a peripheral blood films stained with May-Grünwald Giemsa dyes with cell segmentation (Green mask, all red blood cells) and cellular classifier overlays (Blue mask, reticulocyte; red mask, micronucleus-negative normochromic erythrocyte; green mask, micronucleus-positive normochromic erythrocyte). **B**, Representative image of a peripheral blood films stained with May-Grünwald Giemsa dyes and cell segmentation (Green mask, all red blood cells) and cellular classifier overlays to distinguish morphological abnormalities associated with poor pre-analytical conditions (Blue mask, elongated red blood cell; red mask, healthy normochromic erythrocyte; green mask, reticulocyte; yellow mask, echinocyte).

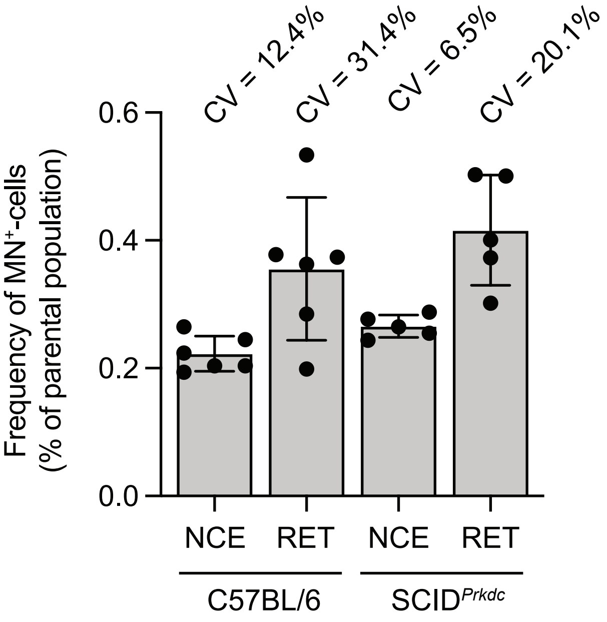

Supplementary Figure 6: Reticulocytes demonstrate higher baseline micronucleus frequencies and greater biological variability than mature erythrocytes. Quantification of the frequency of micronucleus-positive normochromic erythrocytes (MN^+-NCEs) and micronucleus-positive reticulocytes (MN^+-RETs) from treatment-naive C57BL/6 and $SCID^{Prkdc}$ mice by flow cytometry (data are the mean ± s.d.; each point represents one mouse; *n* = 6 C57BL/6 and 5 $SCID^{Prkdc}$ mice; CV = coefficient of variance).

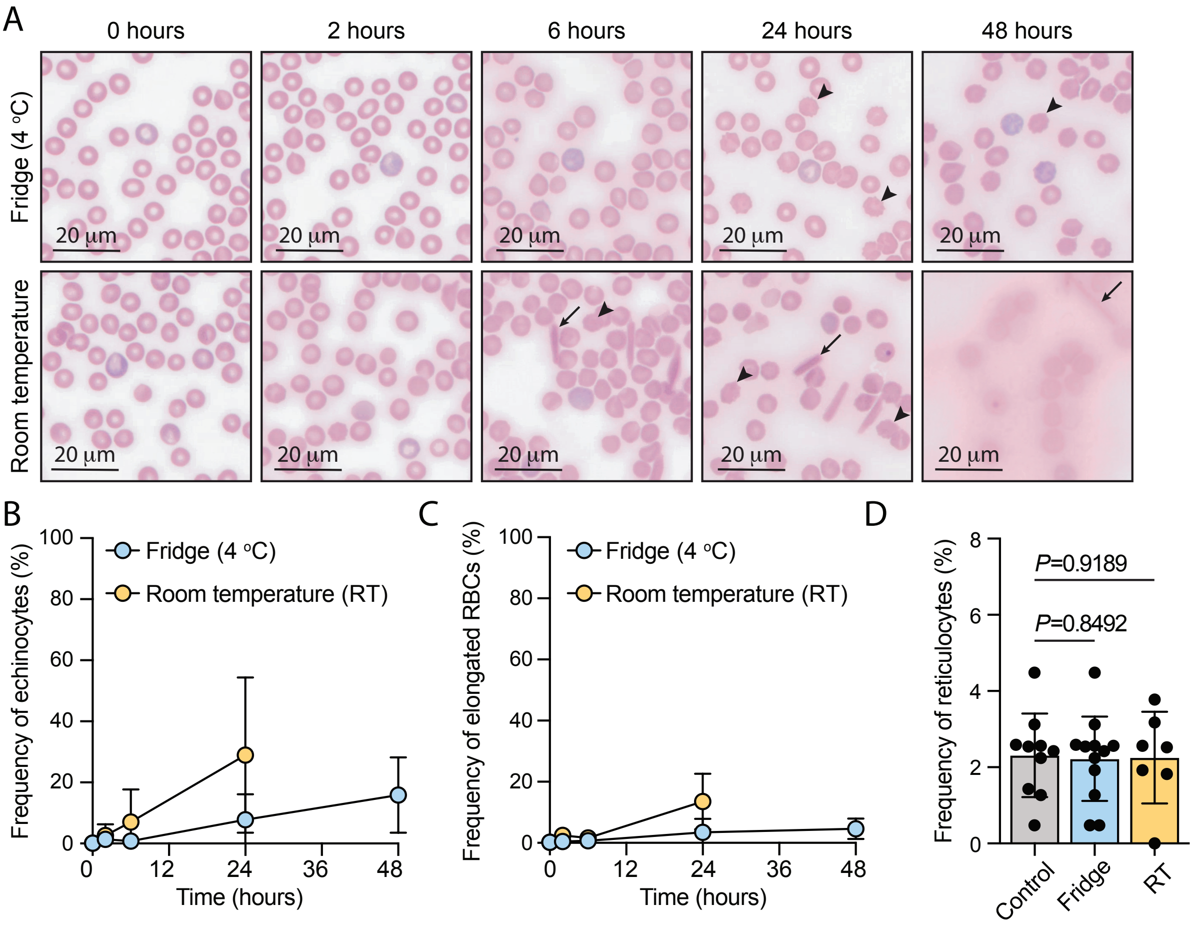

Supplementary Figure 7: Whole-slide imaging allows the algorithmic assessment of morphological features associated with adverse pre-analytical conditions. A, Representative images of peripheral blood smears stained with May-Grünwald Giemsa following pro-longed storage at room temperature or 4°C before blood smear preparation. **B**, Quantification of the frequency of crenated red blood cells displaying membrane spicules (echinocytes) (data are mean ± s.d.; each point represents data from a single mouse; RT, $n = 4$ mice per timepoint ; 4°C, $n = 4$ mice per timepoint). **C**, Quantification of the frequency of red blood cells exhibiting elongated morphologies (data are mean ± s.d.; each point represents data from a single mouse; RT, $n = 4$ mice per timepoint; 4°C, $n = 4$ mice per timepoint). **D**, Quantification of the frequency of reticulocytes from blood samples stored for <24 hours at room temperature (RT) or at 4°C (fridge) and controls (*P*-value calculated using a two-tailed Welch's t-test, data are mean ± s.d.; each point represents data from one mouse, n = 10, 12, and 7, left to right).

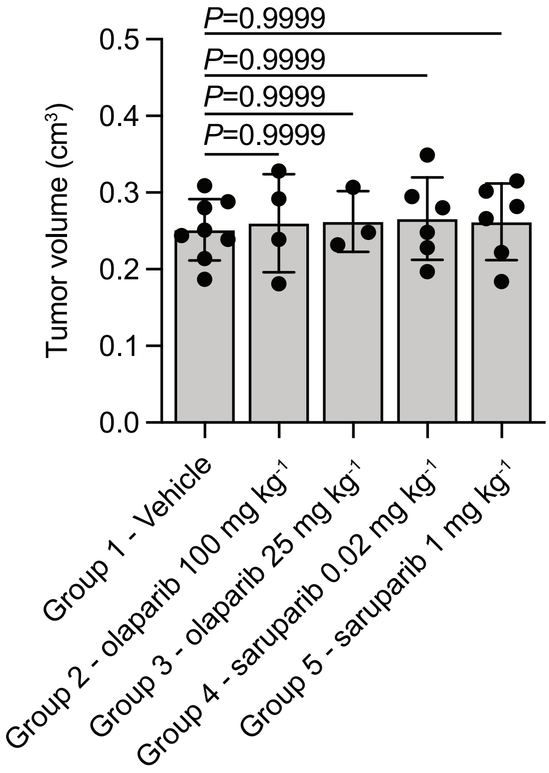

Supplementary Figure 8: Xenograft tumor volumes at randomization across treatment groups. Quantification of individual starting tumor volumes for all MDA-MB-436 xenograft-bearing mice at the point of randomization (*P*-values calculated using the non-parametric Kruskal-Wallis test; the data represent the mean ± s.d.; each point represents data from one mouse, n = 8, 4, 3, 6 and 6, left to right).

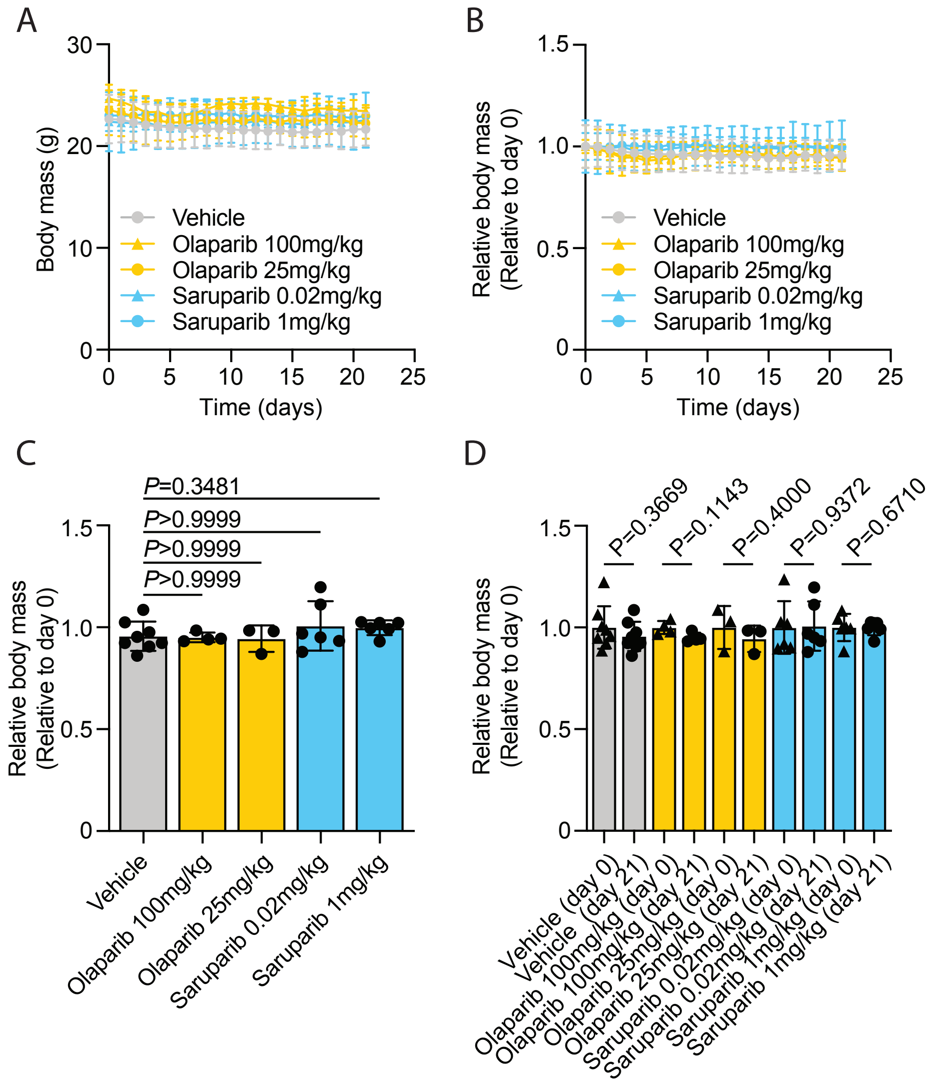

Supplementary Figure 9: Body weight monitoring. A, Raw body weights for all individual mice across all treatment groups (data represent mean ± s.d., *n* = 8 vehicle, 4 olaparib (100 mg kg^{-1}), 3 olaparib (25 mg kg^{-1}), 6 saruparib (0.02 mg kg^{-1}) and 6 saruparib (1 mg kg^{-1}) mice). **B**, Relative body mass normalized to day 0 for each treatment group (data represent mean ± s.d., *n* = 8 vehicle, 4 olaparib (100 mg kg^{-1}), 3 olaparib (25 mg kg^{-1}), 6 saruparib (0.02 mg kg^{-1}) and 6 saruparib (1 mg kg^{-1}) mice). **C**, relative body weight changes observed at day 21 (end of treatment) made relative to day 0 starting weights at randomization (*P*-values calculated using the non-parametric Kruskal-Wallis test, data represent mean ± s.d., *n* = 8 vehicle, 4 olaparib (100 mg kg^{-1}), 3 olaparib (25 mg kg^{-1}), 6 saruparib (0.02 mg kg^{-1}) and 6 saruparib (1 mg kg^{-1}) mice). **D**, relative body mass observed at day 0 and day 21 (end of treatment) made relative to day 0 starting weights at randomization (*P*-values calculated using a two-tailed Mann–Whitney U-test, data represent mean ± s.d., *n* = 8 vehicle, 4 olaparib (100 mg kg^{-1}), 3 olaparib (25 mg kg^{-1}), 6 saruparib (0.02 mg kg^{-1}) and 6 saruparib (1 mg kg^{-1}) mice per group).
